# Deregulation of apolipoprotein C2 gene in cancer: A potential metabolic vulnerability

**DOI:** 10.1002/ctm2.406

**Published:** 2021-05-28

**Authors:** Yuqiao Liu, Yiting Meng, Tian Zhang, Houda Alachkar

**Affiliations:** ^1^ Titus Family Department of Clinical Pharmacy School of Pharmacy, University of Southern California Los Angeles California USA; ^2^ Keck School of Medicine University of Southern California Los Angeles California USA; ^3^ USC Norris Comprehensive Cancer Center University of Southern California Los Angeles California USA

**Keywords:** APOC2, cancer, lipid metabolism

**Abbreviations**
AML: acute myeloid leukemia
APOC2: apolipoprotein C2
FA: fatty acid
OS: overall survival

Dear Editor,

Apolipoprotein C2 (APOC2), an activator of lipoprotein lipase, participates in hydrolysis of triglycerides, very low‐density lipoproteins, and high‐density lipoproteins to release free fatty acids (FA).^1^ FA oxidation has emerged as an important source of energy for cancer survival and growth.^2^ Patterns of *APOC2* genomics and transcriptomic alterations in cancer remained unexplored. Here, we characterize *APOC2* deregulation in cancer by analyzing 176 studies (supplementary‐methods).

In 46706 samples of 34 different cancers (Table S1), amplification, mutation, and deep deletion were the main identified *APOC2* alterations (Figure S1A). Approximately 1% (251 patients) had at least one genetic alteration in *APOC2* with the highest frequency (9.72%) present in bladder cancer and gene amplification being the most common alteration. The highest frequency of deep deletions in *APOC2* occurred in diffuse glioma (1.36%). *APOC2* mutations occurred in 2.3% of non‐melanoma skin cancer and at lower frequencies in other cancers (Table S2). Among the 26 different *APOC2* missense and truncating mutations, two were previously reported in hypertriglyceridemia (Figure S1B; Tables S3 and S4).

*APOC2* was expressed at significantly higher levels in several malignancies (Figures 1 and S2), such as patient‐derived glioblastoma stem cells (33.7‐fold, *p *< 0.0001; Figure 1A), invasive ductal breast cancer (2.9‐fold, *p *< 0.0001; Figure 1B), centroblastic lymphoma (10.1‐fold, *p *< 0.0001; Figure 1C), early stage colorectal tumor (3.8‐fold, *p *= 0.0001; Figure 1D), hypopharyngeal cancer (2.1‐fold, *p *= 0.04; Figure 1E), clear cell renal cell carcinoma (5.9‐fold, *p *< 0.001; Figure 1F), skin squamous cell carcinoma (2.3‐fold, *p *= 0.023; Figure 1G), and gastric cancer (4.8‐fold, *p *= 0.002; Figure 1H) compared with the corresponding normal tissues.

**FIGURE 1:**
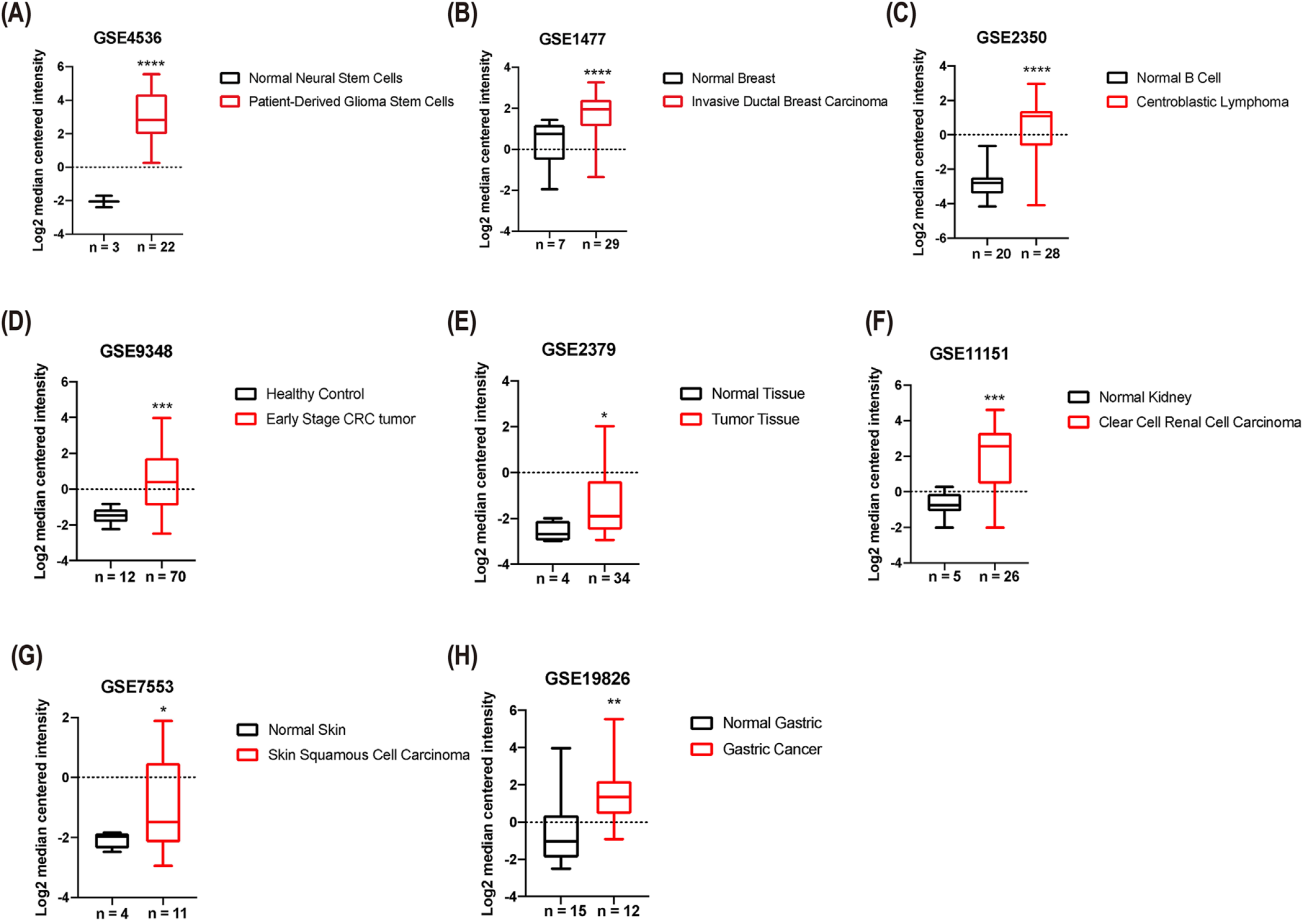
Analysis of *APOC2* expression in different cancers. *APOC2* expression data were retrieved from the GEO database. The mean fold change was compared between cancer group and healthy control in (A) patient‐derived glioma stem cells, (B) invasive ductal breast carcinoma, (C) centroblastic lymphoma, (D) early stage colorectal tumor, (E) hypopharyngeal cancer, (F) clear cell renal cell carcinoma, (G) skin squamous cell carcinoma, and (H) gastric cancer. The differences between groups were analyzed by unpaired t‐test, with the exception of panel (B), (D), and (E), which were analyzed by Mann‐Whitney test. (^****^ *p* < 0.0001, ^***^ *p* < 0.001, ^**^ *p* < 0.01, ^*^ *p* < 0.05)

We also compared *APOC2* DNA methylation beta (β) values corresponding to *APOC2* gene between primary tumors and normal tissues. *APOC2* median β‐values were significantly lower in tumor samples compared with controls (breast invasive carcinoma: 0.809 vs. 0.852; *p *< 0.0001; Figure 2A; colon adenocarcinoma: 0.7 vs. 0.81; *p *< 0.0001; Figure 2B; kidney renal clear cell carcinoma: 0.79 vs. 0.83; *p *< 0.0001; Figure 2C; and stomach adenocarcinoma: 0.676 vs. 0.772; *p *= 0.116; Figure 2D).

**FIGURE 2:**
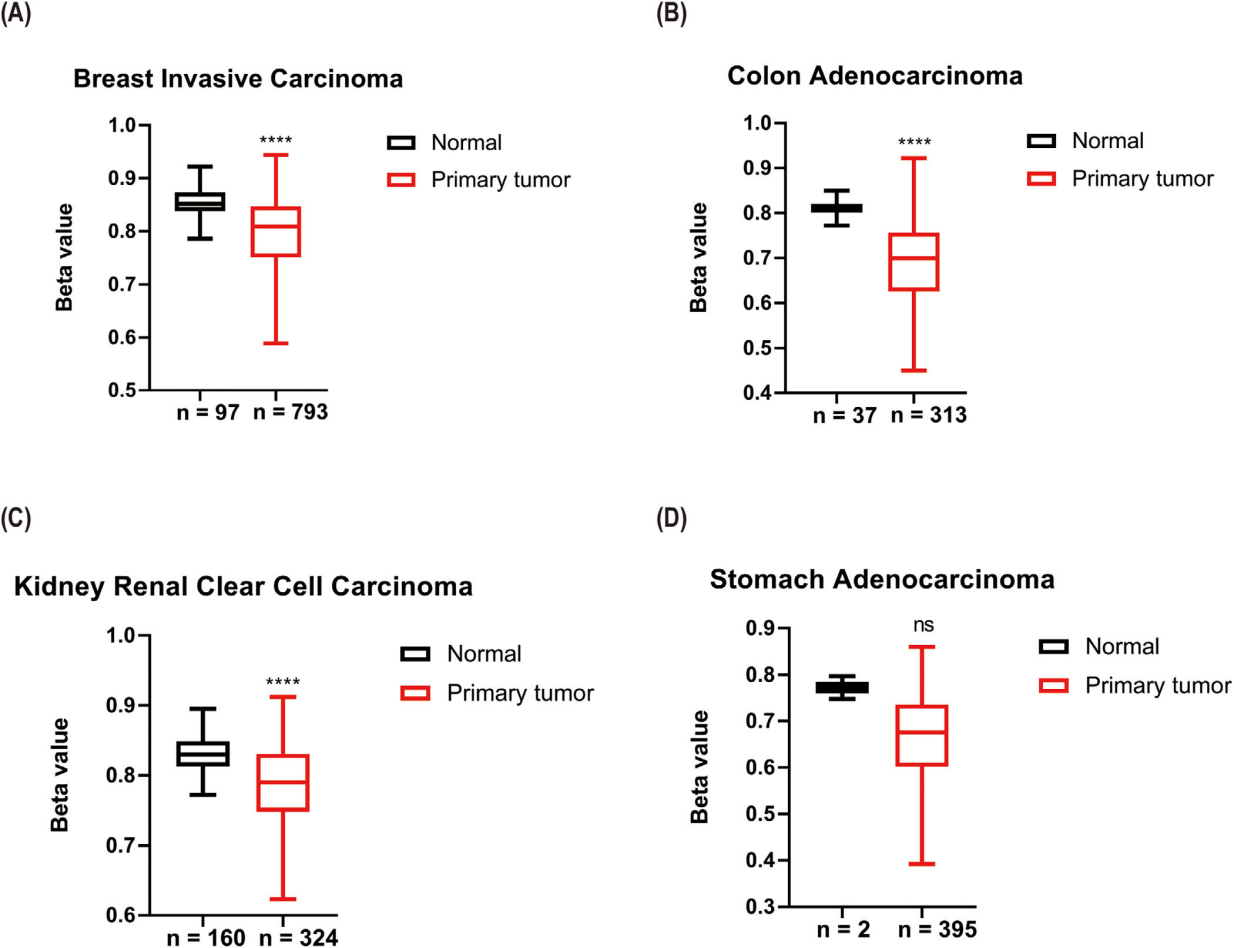
DNA methylation levels of *APOC2* in cancers where *APOC2* is upregulated. Analysis was performed for (A) breast invasive carcinoma, (B) colon adenocarcinoma, (C) kidney renal clear cell carcinoma, and (D) stomach adenocarcinoma. The differences between groups were analyzed by t‐test. (^****^ *p* < 0.0001). Abbreviation: ns, not significant

The association between *APOC2* alteration and chromosomal alterations, clinical attributes, and mutational status are summarized in Figure S3 and Table S5. *APOC2* gene alterations were more frequently found in patients with *TP53, RYR1, RYR2*, and *LRP1B* mutations (*p *< 0.01; Table S6).

We further evaluated cell signaling pathways associated with *APOC2* genetic alterations (amplification, mutation, and high mRNA levels) by ingenuity pathway analysis in several solid malignancies. Several pathways involved in lipid metabolism were altered (the upregulation of phospholipase C signaling and the downregulation of PPAR signaling), and pathways involved in innate and adaptive immune response were also modulated (Figure S4). The dendritic cells maturation pathway, pathways involved in the cross talk between dendritic cells and natural killer cells, Th1 and Th2 activation pathway were upregulated, while the PD‐1 and PD‐L1 cancer immunotherapy pathway was downregulated (Tables S8 and S9).

Survival analysis on all patients from different datasets showed that patients with *APOC2* alterations (mutations and copy number alterations) had significantly shorter median overall survival (OS) compared with patients without alterations (OS: 37.97 vs. 80.68 months; *p *< 0.0001; Figure 3A). Association between altered *APOC2* and poor OS was also observed in several types of cancers (Table S10). In esophageal adenocarcinoma, patients with high *APOC2* expression exhibited shorter OS (13.48 vs. 28.11 months; *p *= 0.0363; Figure 3B). In stomach adenocarcinoma, patients with high *APOC2* had a significantly shorter OS (14.96 vs. 36.00 months; *p *= 0.0072; Figure 3C) than patients with low *APOC2*. In ovarian serous cystadenocarcinoma, patients with *APOC2* mutation or high expression had significantly poorer OS than patients with wild‐type *APOC2* and low gene expression (35.80 vs. 44.51 months; *p *= 0.0058; Figure 3D).

**FIGURE 3:**
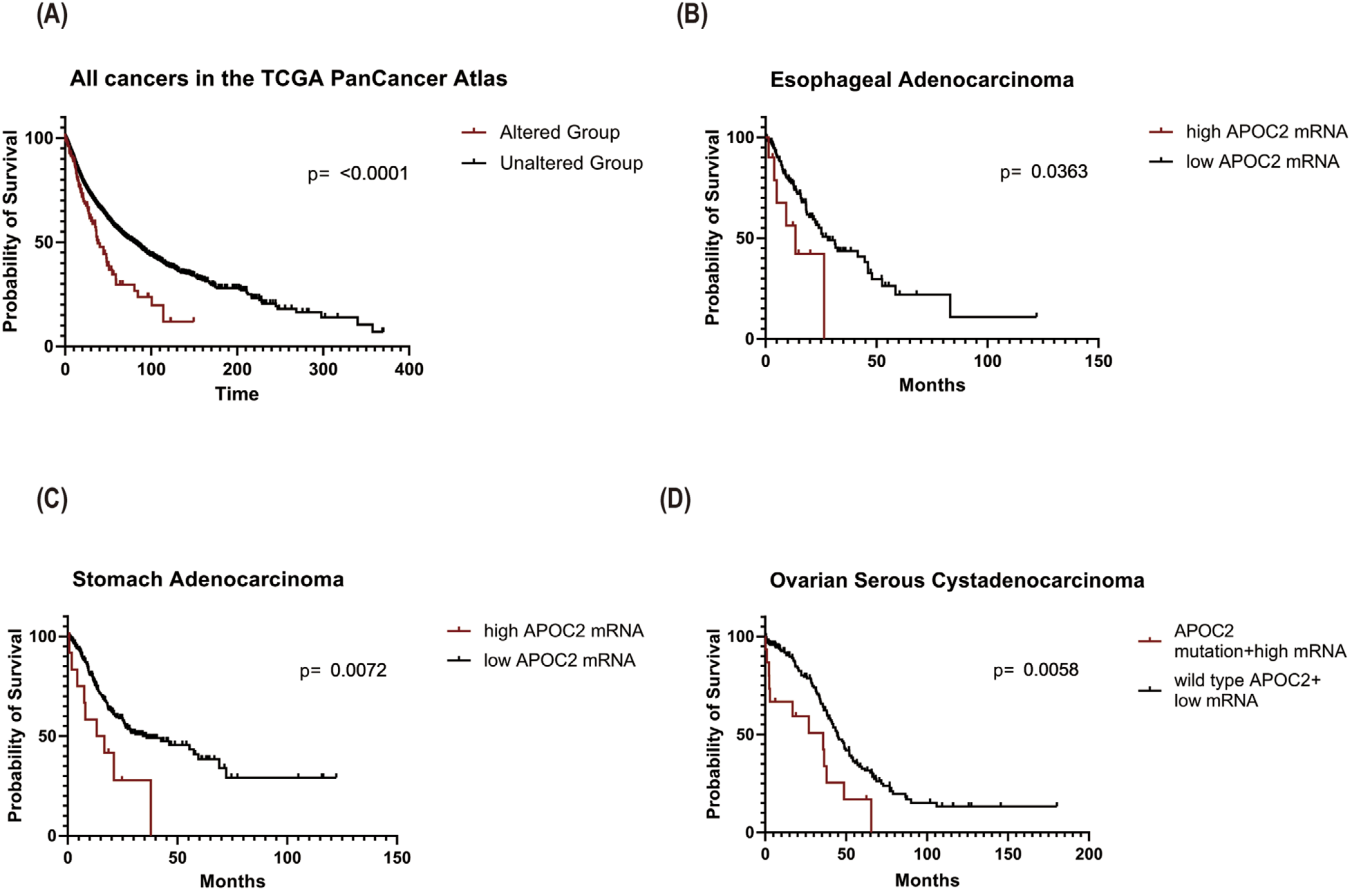
Survival analysis for patients with different cancers associated with *APOC2* alteration and mRNA expression. (A) Overall survival of cancer patients with and without *APOC2* alterations including *APOC2* mutation as well as copy number alterations. (B) Overall survival of esophageal adenocarcinoma patients with high (Z ≥ 2) and low (Z < 2) *APOC2* mRNA level. (C) Overall survival of stomach adenocarcinoma patients with high (Z ≥ 2) and low (Z < 2) *APOC2* mRNA level. (D) Overall survival of ovarian serous cystadenocarcinoma patients with *APOC2* mutations and high (Z ≥ 2) mRNA level versus wild type *APOC2* and low (Z < 2) mRNA level. The differences between groups were analyzed by Mantel‐Cox test.

Metabolic reprogramming is a hallmark of cancer cells to support tumorigenesis and tumor progression. Lipid metabolism is elevated in cancer to satisfy increased energy demands.^2^ Considering APOC2's known function in FA transport and metabolism, its upregulation is a plausible mechanism for cancer adaptation and growth. By analyzing public genomics data, we found that *APOC2* was upregulated and hypomethylated in several types of cancers. We recently reported *APOC2* upregulation and hypomethylation in acute myeloid leukemia (AML) and more notably in AML with mixed‐lineage leukemia (MLL)‐rearrangements suggesting an epigenetic mechanism regulating *APOC2* expression.^3^ Additionally, the transcription factor STAT1 interacts with RXRα on *APOC2* promoter to drive *APOC2* expression in macrophages.^4^ Upregulation of APOC2 in gastrointestinal stromal tumor was associated with cancer cell proliferation, migration, and invasion.^5^ Targeting APOC2 in AML caused antileukemia activity via inhibiting CD36‐ERK pathway.^3^

Consistent with our findings, high APOC2 serum levels were previously found to be associated with shorter survival in pancreatic cancer.^6^ Upregulation of APOC2 was also found in metastatic tumors such as breast invasive carcinoma and fast‐growing lymphoma, suggesting that APOC2 may serve as a prognostic biomarker in cancer.

*APOC2* deregulations are associated with *TP53* mutations. *TP53* mutations are enriched in early clonal stages regardless of tumor type.^7^ Whether *APOC2* upregulation is also an early event in tumor evolution remains to be determined. P53 is also involved in metabolic mechanisms contributing to tumor suppression.^8^ P53 binding to the promoter region of SREBP‐1 represses the expression of SREBP‐1, leading to downregulation of enzymes involved in FA synthesis.^8^ The mechanistic link between *P53* mutations and *APOC2* deregulation remains to be elucidated. APOC2 deregulations are also associated with mutations in LRP1B, a low‐density lipoprotein receptor, and tumor suppressor.^9^

The association between *APOC2* alterations and the deregulation of immune‐related signaling pathways is particularly interesting. As a secreted protein that binds to CD36 receptor, APOC2 may contribute to cancer cells interaction with the immune microenvironment. CD36 mediates the metabolic adaptation which supports regulatory T‐cell survival in tumors.^10^ Whether APOC2 contributes to the metabolic adaptation of intratumoral immune cells remains unknown.

APOC2 gene alterations and upregulation occur frequently in cancer. Functional and mechanistic studies establishing APOC2 as a therapeutic target and expanding these analyses to other apolipoprotein family members are warranted.

## CONFLICT OF INTEREST

The authors declare that there is no conflict of interest that could be perceived as prejudicing the impartiality of the research reported.

## AUTHOR CONTRIBUTIONS

Yuqiao Liu performed the analyses and generated the figures. Yiting Meng performed and wrote the expression analysis. Tian Zhang performed the survival analysis. Yuqiao Liu and Houda Alachkar wrote the manuscript. Houda Alachkar conceived the project, designed the research, and supervised the project.

## DATA AVAILABILITY STATEMENT

The data that support the findings of this study were derived from the following resources available in the public domain:

cBioPortal (https://www.cbioportal.org/)

Oncomine database (https://www.oncomine.org/resource/main.html)

NCBI‐GEO database (https://www.ncbi.nlm.nih.gov/gds)

UALCAN database (http://ualcan.path.uab.edu/analysis.html)

## Supporting information

Supporting Information

## References

[1] Wolska A , Dunbar RL , Freeman LA , et al. Apolipoprotein C‐II: new findings related to genetics, biochemistry, and role in triglyceride metabolism. Atherosclerosis. 2017;267:49–60. 29100061 10.1016/j.atherosclerosis.2017.10.025 PMC5705268

[2] Boroughs LK , DeBerardinis RJ . Metabolic pathways promoting cancer cell survival and growth. Nat Cell Biol. 2015;17:351–359. 25774832 10.1038/ncb3124 PMC4939711

[3] Zhang T , Yang J , Vaikari VP , et al. Apolipoprotein C2 ‐ CD36 promotes leukemia growth and presents a targetable axis in acute myeloid leukemia. Blood Cancer Discov. 2020;1:198–213. 32944714 10.1158/2643-3230.BCD-19-0077 PMC7494214

[4] Trusca VG , Florea IC , Kardassis D , Gafencu AV . STAT1 interacts with RXRα to upregulate ApoCII gene expression in macrophages. PLoS One. 2012;7:e40463. 22808166 10.1371/journal.pone.0040463 PMC3395716

[5] Chen Y , Qin C , Cui X , Geng W , Xian G , Wang Z . miR‐4510 acts as a tumor suppressor in gastrointestinal stromal tumor by targeting APOC2. J Cell Physiol. 2020;235:5711–5721. 31975384 10.1002/jcp.29506

[6] Xue A , Chang JW , Chung L , et al. Serum apolipoprotein C‐II is prognostic for survival after pancreatic resection for adenocarcinoma. Br J Cancer. 2012;107:1883–1891. 23169340 10.1038/bjc.2012.458 PMC3504954

[7] Gerstung M , Jolly C , Leshchiner I , Dentro SC . The evolutionary history of 2,658 cancers. Nature. 2020;578:122–128. 32025013 10.1038/s41586-019-1907-7 PMC7054212

[8] Liu J , Zhang C , Hu W , Feng Z . Tumor suppressor p53 and metabolism. J Mol Cell Biol. 2019;11 284–292. 30500901 10.1093/jmcb/mjy070 PMC6487777

[9] Liu CX , Musco S , Lisitsina NM , Forgacs E , Minna JD , Lisitsyn NA . LRP‐DIT, a putative endocytic receptor gene, is frequently inactivated in non‐small cell lung cancer cell lines. Cancer Res. 2000;60:1961–1967. 10766186

[10] Wang H , Franco F , Tsui YC , et al. CD36‐mediated metabolic adaptation supports regulatory T cell survival and function in tumors. Nat Immunol. 2020;21:298–308. 32066953 10.1038/s41590-019-0589-5 PMC7043937

