# Supplementary material for: Deregulation of apolipoprotein C2 gene in cancer: A potential metabolic vulnerability
Source: Clin Transl Med. 2021 May 28;11(6):e406. doi: 10.1002/ctm2.406 (PMC8161511; doi:10.1002/ctm2.406)
Supplement: Supplementary file 1 — Supporting Information [file CTM2-11-e406-s001.doc]

# **Supplementary material:**

# **Deregulation of Apolipoprotein C2 gene in cancer, a potential metabolic vulnerability**

**Authors:** Yuqiao Liu^1^, Yiting Meng^1^, Tian Zhang^2^, Houda Alachkar^1,3^

**Affiliations:** ^1^Titus Family Department of Clinical Pharmacy, School of Pharmacy,

University of Southern California, Los Angeles, California.

^2^Keck School of Medicine, University of Southern California, Los Angeles, California.

^3^USC Norris Comprehensive Cancer Center, University of Southern California, Los Angeles, California.

**Corresponding author:** Houda Alachkar

**Affiliation:** Titus Family Department of Clinical Pharmacy, School of Pharmacy,

University of Southern California, Los Angeles, California. USC Norris Comprehensive Cancer Center, University of Southern California, Los Angeles, California.

**E-mail:** [alachkar@usc.edu](mailto:alachkar@usc.edu)

**Key Words:** APOC2; cancer; lipid metabolism

**Supplementary Methods:**

*Patient data sets*

Patient’s genetic alterations and clinical data were downloaded from cBioPortal^1,2^ (https://www.cbioportal.org/) on April 8, 2020. We analyzed data in 176 cancer studies that were manually curated including The Cancer Genome Atlas (TCGA) and non-TCGA studies with 46706 non-overlapping samples. Survival data were downloaded from the TCGA database available at cBioPortal. Patient’s *APOC2* expression data in different types of cancer were downloaded from the Oncomine database ([https://www.oncomine.org/resource/main.html](https://www.oncomine.org/resource/main.html#a%3A2437%3Bd%3A60919987%3Bdso%3AgeneOverex%3Bdt%3ApredefinedClass%3Bec%3A%5B%5D%3Bet%3Aover%3Bf%3A61131947%3Bg%3A344%3Bgt%3Aboxplot%3Bp%3A200001641%3Bpg%3A1%3Bpvf%3A5033%2C150004%3Bscr%3Adatasets%3Bss%3Aanalysis%3Bth%3Ag10.0%2Cp0.050%2Cfc2.0%3Bv%3A18)). Patient’s gene methylation data were downloaded from TCGA analysis available at the UALCAN database^3^ (http://ualcan.path.uab.edu/analysis.html).

*Statistical analysis*

Overall survival (OS) was defined as time between initial diagnosis and death from any reason. Kaplan-Meier survival curves were generated for the comparison of OS between patients with *APOC2* alterations including mutation as well as copy number alterations and those without, and between patients with high (Z > 2) and low (Z < -2) *APOC2* expression level. P-values for OS were calculated using the Mantel-Cox test in GraphPad Prism 8.0 (GraphPad Software, Inc.). P-values for clinical attributes associated with chromosomal gain and loss were calculated using Chi-squared test. Kruskal-Wallis test was used to calculate p-values for clinical attributes associated with fraction genome altered, mutation count and MSIsensor score. P-values for *APOC2* alteration association with gene mutations were calculated by Fisher exact test. Student’s t-test was used to calculate the p-values for *APOC2* methylation. The data in gene expression analysis and DNA methylation analysis were log transformed and most of them were normally distributed. Mann-Whitney test was used for the *APOC2* gene expression data in invasive ductal breast carcinoma, early stage colorectal tumor and hypopharyngeal cancer, which were not normally distributed. P-value less than 0.05 was considered statistically significant.

*Gene expression analysis*

*APOC2* gene expression levels in various cancer types were obtained from the Oncomine database. The significantly overexpressed *APOC2* in cancer tissues compared to normal tissues was identified in Oncomine datasets with setting the threshold parameters by *P*< 0.05, fold-change >2, and gene rank in the top 10%. For each specific cancer study that was identified on Oncomine, *APOC2* expression raw data were retrieved from the NCBI-GEO database of cancer types vs. healthy tissues shown in Table S11. The gene microarray data were analyzed using the GEO query package in R^4^, also all data omitting negative values were log (base 2) transformed and median-centered normalized using the DescTools package in R. The mean fold change was compared in the cancer group and healthy control, and we used an unpaired t-test in R to calculate the p-value for each test.

*DNA methylation analysis*

*APOC2* promoter methylation data of 2121 samples in breast invasive carcinoma, colon adenocarcinoma, kidney renal clear cell carcinoma and stomach adenocarcinoma in TCGA datasets were downloaded from the UALCAN database^3^. The gene symbol “APOC2” was searched and methylation analysis was queried. Beta (β) value is the ratio of the methylated probe intensity and the sum of methylated and unmethylated probe intensity. Beta (β) value indicates level of DNA methylation ranging from 0 (unmethylated) to 1 (fully methylated). We compared methylation beta (β) values associated with probes that correspond to *APOC2* gene between primary tumors and normal tissues. The ID of the probes in Illumina Infinium Human methylation 450 BeadChip were cg22164781, cg27436184, cg10169327, cg14723423 and cg01958934.

*Ingenuity Pathway analysis*

From cBioPortal, we queried samples with *APOC2* mutation, copy number alterations as well as mRNA expression z-scores threshold of ±2 in cancers where *APOC2* was upregulated. Ingenuity pathway analysis (IPA) (QIAGEN Inc., https://digitalinsights.qiagen.com/products-overview/discovery-insights-portfolio/analysis-and-visualization/qiagen-ipa/) was used to identify potential signaling pathways. The expression log ratio of the significant genes in these cancers were imported into IPA. We used comparison analysis to evaluate common pathways that were associated with *APOC2* alterations and high mRNA level.

**Supplementary Tables:**

**Table S1. Clinical characteristics of the patient samples on cBioPortal**

| Total | |
| --- | --- |
| Diagnosis age, y (%) |  |
| ≤5 | 2864 (6.4) |
| 5-20 | 2807 (6.3) |
| 20-35 | 975(2.2) |
| 36-50 | 3016 (6.7) |
| 50-65 | 6370 (14.2) |
| 65-80 | 5423 (12.1) |
| ≥80 | 813 (1.8) |
| NA | 22478 (50.3) |
| Sex, No. (%) |  |
| Female | 20224(45.2) |
| Male | 19825 (44.3) |
| NA | 4702 (10.5) |
| Race, No. (%) |  |
| White/Caucasian | 11499 (25.7) |
| Black/African American | 1325 (3.0) |
| Asian | 797 (1.8) |
| Other/NA | 31134 (69.5) |

**Table S2. *APOC2* gene deregulation in cancer**

| **Cancer type** | **Frequency** | | | **Total case number** |
| --- | --- | --- | --- | --- |
|  | **Amplification** | **Mutation** | **Deep deletion** |  |
| Bladder Cancer | 9.72%  (7 cases) | — | — | 72 (9.72%) |
| Endometrial Carcinoma | 2.56%  (15 cases) | 0.51%  (3 cases) | 0.17%  (1 case) | 586 (3.24%) |
| Cervical Squamous Cell Carcinoma | 3.19%  (8 cases) | — | — | 251 (3.19%) |
| Pancreatic Adenocarcinoma | 2.72%  (5 cases) | — | — | 184 (2.72%) |
| Ovarian Epithelial Tumor | 1.88%  (11 cases) | — | 0.68%  (4 cases) | 584 (2.57%) |
| Pancreatic Cancer | 2.36%  (16 cases) | — | — | 678 (2.36%) |
| Skin Cancer, Non-Melanoma | — | 2.3%  (4 cases) | — | 174 (2.3%) |
| Prostate Cancer | 2.03%  (13 cases) | — | — | 639 (2.03%) |
| Adrenocortical Carcinoma | 2.02%  (2 cases) | — | — | 99 (2.02%) |
| Sarcoma | 1.18%  (3 cases) | — | 0.78%  (2 cases) | 255 (1.96%) |
| Soft Tissue Sarcoma | 1.85%  (7 cases) | — | — | 379 (1.85%) |
| Diffuse Glioma | 0.39%  (2 cases) | — | 1.36%  (7 cases) | 514 (1.75%) |
| Bladder Urothelial Carcinoma | 1.46%  (6 cases) | 0.24%  (1 case) | — | 411 (1.7%) |
| Esophagogastric Adenocarcinoma | 1.35%  (8 cases) | 0.34%  (2 cases) | — | 592 (1.69%) |
| Non-Small Cell Lung Cancer | 1.11%  (21 cases) | 0.32%  (6 cases) | 0.11%  (2 cases) | 1899 (1.53%) |
| Salivary Cancer | 0.94%  (2 cases) | — | 0.47%  (1 case) | 212 (1.42%) |
| Prostate Adenocarcinoma | 0.87%  (10 cases) | — | 0.52%  (6 cases) | 1151 (1.39%) |
| Hepatocellular Carcinoma | 1.36%  (5 cases) | — | — | 369 (1.36%) |
| Invasive Breast Carcinoma | 0.91%  (10 cases) | 0.09%  (1 case) | 0.18%  (2 cases) | 1096 (1.19%) |
| Pleural Mesothelioma | 1.15%  (1 case) | — | — | 87 (1.15%) |
| Wilms Tumor | 1.06%  (2 cases) | — | — | 188 (1.06%) |
| Colon Adenocarcinoma | 0.94%  (1 case) | — | — | 106 (0.94%) |
| Breast Cancer | 0.78%  (24 cases) | 0.03%  (1 case) | 0.1%  (3 cases) | 3090 (0.91%) |
| Head and Neck Squamous Cell Carcinoma | 0.76%  (4 cases) | — | — | 523 (0.76%) |
| Melanoma | 0.23%  (3 cases) | 0.15%  (2 cases) | 0.23%  (3 cases) | 1318 (0.61%) |
| Hepatobiliary Cancer | 0.13%  (1 case) | 0.26%  (2 cases) | — | 777 (0.39%) |
| B-Lymphoblastic Leukemia/Lymphoma | — | — | 0.32%  (3 cases) | 939 (0.32%) |
| Salivary Gland Cancer | 0.21%  (2 cases) | — | 0.11%  (1 case) | 949 (0.32) |
| Bone Cancer | — | 0.31%  (1 case) | — | 324 (0.31%) |
| Renal Clear Cell Carcinoma | 0.2%  (1 case) | — | — | 511 (0.2%) |
| Glioblastoma | 0.17%  (1 case) | — | — | 593 (0.17%) |
| Leukemia | 0.08%  (1 case) | — | 0.08%  (1 case) | 1221 (0.16%) |
| Colorectal Adenocarcinoma | — | 0.08%  (1 case) | — | 1213 (0.08%) |
| Mature B-Cell Neoplasms | — | 0.04%  (1 case) | 0.04%  (1 case) | 2800 (0.07%) |

**Table S3. *APOC2* mutations associated with hypertriglyceridemia**

| ApoCII Variant | Mutation Type | Nucleotide Change | Protein Change |
| --- | --- | --- | --- |
| ApoCII (-190T>A) | Promoter | c.-190T>A | N/A |
| ApoCII (-86A>G) | Promoter | c.-86A>G | N/A |
| ApoCII (PARIS1) | Missense | c.1A>G | M1V |
| ApoCII (PARIS2, BARCELONA) | Nonsense | c.10C>T | R4* |
| ApoCII (JAN, VEN) | FS del | c.70delC | fsQ24 |
| ApoCII (SHANGHAI) | Del-ins | c.86A>CC | fsD29 |
| ApoCII (NIJMEGEN) | FS del | c.118delG | fsV40* |
| ApoCII variant   (ApoCII-v) | Missense | c.122A>C | K41T |
| ApoCII (WAKAYAMA) | Missense | c.142T>C | W48R |
| ApoCII (BARI) | Nonsense | c.177C>G | Y59* |
| ApoCII (PADOVA) | Nonsense | c.177C>A | Y59* |
| ApoCII (SAN FRANCISCO) | Missense | c.178G>A | E60K |
| ApoCII (PHILADELPHIA) | Missense | c.215G>C | R72T |
| ApoCII (AFRICAN) | Missense | c.229A>C | K77Q |
| ApoCII (AUCKLAND) | Nonsense | c.255C>A | Y85* |
| ApoCII (TORONTO) | FS del | c.270delT | fsT90 |
| ApoCII (ONTARIO) | Nonsense | c.274C>T | Q92* |
| ApoCII (ST. MICHAEL) | FS ins | c.274dupC | fsQ92 |
| ApoCII (HONGKONG) | Missense | c.281T>C | L94P |
| ApoCII (HAM, TOK) | Splice | c.55+1G>C | Splice NT 2 G+1 to C |
| ApoCII (TUZLA) | Deletion | Loss of Ex 2,3,4 | untranslated |
| ApoCII (COLOMBIA) | FS del | c.133_134delTC | fsS67 |

**Table S4. *APOC2* mutations associated with cancer**

| Cancer Type | Mutation Type | Nucleotide Change | Protein Change |
| --- | --- | --- | --- |
| Stomach Adenocarcinoma | Missense | c.242C>A | A81D |
| Tubular Stomach Adenocarcinoma | FS del | c.25_26del | L9Vfs*10 |
| Lung Adenocarcinoma | Missense | c.257C>A | T86K |
| Lung Adenocarcinoma | Nonstop | c.305A>T | *102Lext*23 |
| Lung Squamous Cell Carcinoma | Missense | c.128C>G | S43C |
| Lung Squamous Cell Carcinoma | Missense | c.156G>C | K52N |
| Lung Adenocarcinoma | Missense | c.100C>G | P34A |
| Bladder Urothelial Carcinoma | Nonsense | c.10C>T | R4* |
| Cutaneous Melanoma | Missense | c.100C>T | P34S |
| Hepatocellular Carcinoma | Missense | c.26T>C | L9P |
| Hepatocellular Carcinoma | Missense | c.202G>T | D68Y |
| Melanoma | Missense | c.5G>A | G2D |
| Cutaneous Squamous Cell Carcinoma | Missense | c.295G>A | G99R |
| Skin Cancer, Non-Melanoma | Missense | c.295G>A | G99R |
| Breast Invasive Ductal Carcinoma | Splice |  | X71_splice |
| Colon Adenocarcinoma | Missense | c.163G>A | A55T |
| Skin Cancer, Non-Melanoma | Missense | c.295_296delinsAA | G99K |
| Skin Cancer, Non-Melanoma | Missense | c.124G>A | E42K |
| Osteosarcoma | Missense | c.58G>A | V20I |
| Lung Adenocarcinoma | Missense | c.100C>A | P34T |
| Germinal Center B-cell Like Diffuse Large B-cell Lymphoma | Missense | c.122A>C | K41T |
| Breast Invasive Carcinoma | FS del | c.166del | Q56Rfs*12 |
| Lung Adenocarcinoma | Splice | c.216-3C>T | X72_splice |
| Uterine Serous Carcinoma/Uterine Papillary Serous Carcinoma | Missense | c.197C>A | A66D |
| Uterine Endometrioid Carcinoma | Missense | c.265T>C | F89L |
| Uterine Endometrioid Carcinoma | Missense | c.253T>C | Y85H |

**Table S5. Chromosomal status associated with alteration of *APOC2***

| **Chromosomal Status** | **p-Value** | **Frequency in altered group**  **% (N)** | | | | | **Frequency in unaltered group**  **% (N)** | | |
| --- | --- | --- | --- | --- | --- | --- | --- | --- | --- |
|  |  | **Gained** | **Lost** | | **Not Called** | **Gained** | | **Lost** | **Not Called** |
| 19q Status | <10^-10^ | 50% (29) | | 10.34% (6) | 39.66% (23) | | 13.01% (1157) | 10.87% (966) | 76.12% (6767) |
| 10p Status | 3.46E-10 | 28.7% (33) | | 15.65% (18) | 55.65% (64) | | 9.96% (947) | 19.77% (1879) | 70.27% (6678) |
| 19p Status | 4.31E-7 | 10.23% (9) | | 32.95% (29) | 56.82% (50) | | 9.1% (809) | 13.43% (1194) | 77.47% (6887) |
| 15 (15q) Status | 9.43E-7 | 6.67% (7) | | 38.1% (40) | 55.24% (58) | | 5.26% (470) | 18.48% (1652) | 76.26% (6815) |
| 12p Status | 1.39E-6 | 33.63% (38) | | 13.27% (15) | 53.1% (60) | | 17.5% (1635) | 8.07% (754) | 74.43% (6953) |
| 16p Status | 4.64E-6 | 14.29% (16) | | 24.11% (27) | 61.61% (69) | | 13.73% (1304) | 10.05% (954) | 76.22% (7237) |
| 22 (22q) Status | 1.57E-5 | 9.8% (10) | | 43.14% (44) | 47.06% (48) | | 6.89% (637) | 24.56% (2270) | 68.55% (6337) |
| 17p Status | 5.35E-5 | 3.51% (4) | | 55.26% (63) | 41.23% (47) | | 5.22% (497) | 35.27% (3360) | 59.52% (5670) |
| 5p Status | 6.22E-4 | 29.66% (35) | | 13.56% (16) | 56.78% (67) | | 23.84% (2279) | 6.16% (589) | 69.99% (6690) |
| 2p Status | 1.60E-3 | 20.95% (22) | | 1.9% (2) | 77.14% (81) | | 11.13% (1045) | 6.75% (634) | 82.12% (7710) |
| 12q Status | 2.04E-3 | 18.75% (21) | | 14.29% (16) | 66.96% (75) | | 11.03% (1005) | 8.66% (789) | 80.31% (7315) |
| 4q Status | 2.35E-3 | 1.79% (2) | | 37.5% (42) | 60.71% (68) | | 3.11% (282) | 23.55% (2138) | 73.34% (6657) |
| 5q Status | 3.12E-3 | 5.66% (6) | | 35.85% (38) | 58.49% (62) | | 7.91% (706) | 22.1% (1973) | 69.99% (6247) |
| 9p Status | 4.02E-3 | 14.29% (17) | | 31.93% (38) | 53.78% (64) | | 7.06% (637) | 28.68% (2589) | 64.27% (5802) |
| 3q Status | 4.23E-3 | 32.71% (35) | | 5.61% (6) | 61.68% (66) | | 19.92% (1773) | 7.89% (702) | 72.19% (6424) |
| 1q Status | 4.91E-3 | 41.82% (46) | | 1.82% (2) | 56.36% (62) | | 28.28% (2596) | 4.43% (407) | 67.29% (6178) |
| 4p Status | 5.29E-3 | 5.04% (6) | | 35.29% (42) | 59.66% (71) | | 4.87% (467) | 22.86% (2192) | 72.26% (6928) |
| 20q Status | 7.08E-3 | 38.74% (43) | | 5.41% (6) | 55.86% (62) | | 29.63% (2822) | 2.32% (221) | 68.05% (6482) |

**Table S6. Genes with highest mutation frequencies in *APOC2* altered and unaltered groups**

| **Gene** | **Altered (n = 132)**  **N (%)** | **Unaltered (n = 10305)**  **N (%)** | **p-Value** |
| --- | --- | --- | --- |
| TP53 | 78 (59.09%) | 3761 (36.50%) | 1.36E-7 |
| TTN | 50 (37.88%) | 3086 (29.95%) | 0.0319 |
| MUC16 | 43 (32.58%) | 1971 (19.13%) | 1.88E-4 |
| CSMD3 | 32 (24.24%) | 1318 (12.79%) | 2.68E-4 |
| RYR2 | 31 (23.48%) | 1289 (12.51%) | 4.04E-4 |
| USH2A | 27 (20.45%) | 1103 (10.70%) | 7.97E-4 |
| LRP1B | 27 (20.45%) | 1270 (12.32%) | 5.79E-3 |
| FLG | 25 (18.94%) | 1146 (11.12%) | 5.85E-3 |
| RYR1 | 23 (17.42%) | 841 (8.16%) | 4.78E-4 |
| XIRP2 | 23 (17.42%) | 874 (8.48%) | 8.05E-4 |

**Table S7. *TP53* mutation in *APOC2* altered and unaltered groups in cancer**

| **Cancer Type** | **Altered** | **Unaltered** | **p-Value** |
| --- | --- | --- | --- |
| Breast Cancer | 46.74% (43/92) | 34.00% (616/1812) | 9.23E-03 |
| Colorectal Adenocarcinoma | 78.13% (25/32) | 58.54% (288/492) | 0.02 |
| Brain Lower Grade Glioma | 69.57% (16/23) | 48.14% (233/484) | 0.036 |
| Pancreatic Adenocarcinoma | 88.89% (8/9) | 59.12% (94/159) | 0.071 |
| Skin Cutaneous Melanoma | 33.33% (6/18) | 16.81% (58/345) | 0.077 |

**Table S8. z-scores of Ingenuity Pathway Analysis results**

| **Canonical Pathways** | **Breast** | **Cervical** | | **HNSC** | | **BLCA** |
| --- | --- | --- | --- | --- | --- | --- |
| Dendritic Cell Maturation | 5.75 | 5.396 | 5.209 | | 4.841 | |
| Role of NFAT in Regulation of the Immune Response | 5.093 | 5.745 | 5.425 | | 4.768 | |
| Crosstalk between Dendritic Cells and Natural Killer Cells | 5.396 | 5.385 | 5 | | 4.472 | |
| PKCθ Signaling in T Lymphocytes | 5.259 | 5.014 | 5.657 | | 4.315 | |
| Th1 Pathway | 4.644 | 5.568 | 5.396 | | 4.017 | |
| Neuroinflammation Signaling Pathway | 4.781 | 4.667 | 4.459 | | 5.345 | |
| iCOS-iCOSL Signaling in T Helper Cells | 3.618 | 4.914 | 5.292 | | 4.379 | |
| TREM1 Signaling | 5.568 | 3.873 | 4.243 | | 4.123 | |
| Production of Nitric Oxide and Reactive Oxygen Species in Macrophages | 4.429 | 4.2 | 4.899 | | 3.53 | |
| Systemic Lupus Erythematosus In T Cell Signaling Pathway | 2.528 | 4.95 | 4.564 | | 4.218 | |
| IL-8 Signaling | 3.015 | 3.71 | 3.9 | | 4.243 | |
| Natural Killer Cell Signaling | 4.041 | 3.812 | 4.629 | | 2.271 | |
| Phospholipase C Signaling | 2.794 | 3.71 | 4.472 | | 3.528 | |
| PD-1, PD-L1 cancer immunotherapy pathway | -2.53 | -4.271 | -3.651 | | -4.041 | |
| Tec Kinase Signaling | 2.535 | 3.9 | 4.379 | | 3.578 | |
| CREB Signaling in Neurons | 1.2 | 1.857 | 5.24 | | 5.963 | |
| Calcium-induced T Lymphocyte Apoptosis | 3.128 | 3.5 | 4.123 | | 3.464 | |
| Breast Cancer Regulation by Stathmin1 | 2.55 | 1.633 | 4.323 | | 5.455 | |
| Fcγ Receptor-mediated Phagocytosis in Macrophages and Monocytes | 3.212 | 3.273 | 3.742 | | 3.441 | |
| IL-17 Signaling | 4.003 | 3.606 | 3.638 | | 2.4 | |
| Integrin Signaling | 2.188 | 2.982 | 3.771 | | 4.7 | |
| Leukocyte Extravasation Signaling | 2.714 | 4.041 | 3.962 | | 2.921 | |
| Type I Diabetes Mellitus Signaling | 4.849 | 3.207 | 3.606 | | 1.89 | |
| Inhibition of ARE-Mediated mRNA Degradation Pathway | 3.838 | 3.464 | 3.317 | | 2.84 | |
| Role of Pattern Recognition Receptors in Recognition of Bacteria and Viruses | 3.651 | 3.207 | 3.606 | | 2.84 | |
| CD28 Signaling in T Helper Cells | 3.124 | 3.578 | 3.578 | | 2.982 | |
| HIF1α Signaling | 2.846 | 3.5 | 2.673 | | 3.4 | |
| Cardiac Hypertrophy Signaling (Enhanced) | 2.288 | 2.137 | 4.459 | | 3.515 | |
| IL-15 Production | 2.959 | 3.357 | 3.771 | | 2.294 | |
| Colorectal Cancer Metastasis Signaling | 1.604 | 3.153 | 3.9 | | 3.656 | |
| Regulation Of The Epithelial Mesenchymal Transition By Growth Factors Pathway | 1.897 | 2.668 | 3.578 | | 4.017 | |
| Chemokine Signaling | 3.53 | 3 | 2.887 | | 2.714 | |
| IL-15 Signaling | 2.837 | 3.317 | 3.207 | | 2.714 | |
| Hepatic Fibrosis Signaling Pathway | 2.429 | 3.13 | 3.138 | | 3.207 | |
| B Cell Receptor Signaling | 3.098 | 2 | 3 | | 3.578 | |
| Ephrin Receptor Signaling | 2.596 | 1.897 | 2.84 | | 4.264 | |
| Th2 Pathway | 3 | 3.024 | 3.024 | | 2.414 | |
| Systemic Lupus Erythematosus In B Cell Signaling Pathway | 3.215 | 2.846 | 3.162 | | 2.16 | |
| PPAR Signaling | -4.49 | -2 | -2.53 | | -2.324 | |

**Table S9. -log(B-H p-value) of Ingenuity Pathway Analysis results**

| **Canonical Pathways** | **Breast** | **Cervical** | **HNSC** | **BLCA** |
| --- | --- | --- | --- | --- |
| Th1 and Th2 Activation Pathway | 13.96854 | 24.4442 | 33.01883 | 13.72257 |
| Th1 Pathway | 12.55284 | 21.59075 | 30.07198 | 12.5141 |
| Th2 Pathway | 10.96492 | 22.07059 | 26.5514 | 12.5141 |
| iCOS-iCOSL Signaling in T Helper Cells | 16.76354 | 16.2991 | 21.58176 | 6.322849 |
| Natural Killer Cell Signaling | 15.82531 | 15.37647 | 19.55529 | 4.100786 |
| PD-1, PD-L1 cancer immunotherapy pathway | 10.62646 | 15.33793 | 20.21685 | 7.184974 |
| CD28 Signaling in T Helper Cells | 13.78833 | 13.47955 | 19.31728 | 5.484102 |
| Role of NFAT in Regulation of the Immune Response | 17.27199 | 9.831735 | 16.81221 | 6.650397 |
| T Cell Exhaustion Signaling Pathway | 11.39984 | 12.99322 | 17.31697 | 6.648047 |
| Dendritic Cell Maturation | 14.90541 | 11.62038 | 13.39921 | 7.184974 |
| Crosstalk between Dendritic Cells and Natural Killer Cells | 12.98429 | 15.78971 | 13.44477 | 4.100786 |
| Altered T Cell and B Cell Signaling in Rheumatoid Arthritis | 14.12739 | 9.631984 | 17.31697 | 4.521195 |
| T Helper Cell Differentiation | 8.37569 | 11.6564 | 15.6229 | 5.559847 |
| Antigen Presentation Pathway | 10.98603 | 12.70031 | 10.15273 | 6.940501 |
| Communication between Innate and Adaptive Immune Cells | 11.64356 | 9.77544 | 13.52918 | 4.521195 |
| CTLA4 Signaling in Cytotoxic T Lymphocytes | 9.560024 | 13.07387 | 13.44477 | 2.779854 |
| PKCθ Signaling in T Lymphocytes | 13.38516 | 8.453346 | 13.97238 | 2.421 |
| Type I Diabetes Mellitus Signaling | 14.12739 | 9.831735 | 11.13655 | 2.062291 |
| Systemic Lupus Erythematosus In B Cell Signaling Pathway | 15.31973 | 7.577574 | 11.39626 | 2.191165 |
| T Cell Receptor Signaling | 13.16484 | 6.773308 | 12.51227 | 1.94637 |
| Phagosome Formation | 8.543273 | 8.061541 | 8.708587 | 8.980028 |
| Graft-versus-Host Disease Signaling | 8.37569 | 8.779995 | 10.85165 | 5.484102 |
| Neuroinflammation Signaling Pathway | 7.168107 | 7.492774 | 9.679055 | 7.170884 |
| Autoimmune Thyroid Disease Signaling | 5.953169 | 9.631984 | 10.70196 | 4.652253 |
| Pathogenesis of Multiple Sclerosis | 5.060983 | 10.16551 | 7.365341 | 7.285324 |
| Allograft Rejection Signaling | 4.062415 | 8.453346 | 10.93531 | 3.366138 |
| B Cell Development | 4.389133 | 6.835779 | 8.435663 | 5.163868 |
| Role of Pattern Recognition Receptors in Recognition of Bacteria and Viruses | 6.844017 | 5.570442 | 8.794573 | 1.908191 |
| OX40 Signaling Pathway | 3.697071 | 7.318297 | 8.815089 | 3.112476 |
| IL-4 Signaling | 8.319988 | 4.954016 | 6.83618 | 2.618265 |
| Calcium-induced T Lymphocyte Apoptosis | 5.209927 | 6.608053 | 9.434611 | 1.451488 |
| Tec Kinase Signaling | 5.280867 | 5.172187 | 10.42861 | 1.817021 |
| TREM1 Signaling | 7.399895 | 4.335155 | 8.516418 | 2.437071 |
| Granulocyte Adhesion and Diapedesis | 8.170097 | 5.172187 | 4.237728 | 4.630398 |
| Phospholipase C Signaling | 9.68848 | 4.308972 | 5.122313 | 2.913021 |
| Leukocyte Extravasation Signaling | 4.369033 | 6.845884 | 7.365341 | 3.287256 |
| Production of Nitric Oxide and Reactive Oxygen Species in Macrophages | 5.935789 | 5.449946 | 8.815089 | 1.202467 |
| Primary Immunodeficiency Signaling | 5.794237 | 6.73663 | 7.486297 | 0.898645 |
| Atherosclerosis Signaling | 3.2687 | 3.704161 | 6.963551 | 6.940501 |

**Table S10. Association between *APOC2* gene expression, alteration and cancer patient survival**

| **Cancer type** | **Survival**  **(p-Value)** | **Number of altered samples / total number of samples** | |
| --- | --- | --- | --- |
| Colorectal Adenocarcinoma | 0.562 | | 36/594 |
| Breast Invasive Carcinoma | 0.346 | | 52/994 |
| Glioblastoma Multiforme | 0.974 | | 5/378 |
| Cervical Squamous Cell Carcinoma | 0.111 | | 20/275 |
| Esophageal Adenocarcinoma | 0.0363 | | 10/181 |
| Stomach Adenocarcinoma (mutation + high mRNA) | 0.162 | | 22/407 |
| Stomach Adenocarcinoma (high mRNA only) | 0.0072 | | 13/407 |
| Stomach Adenocarcinoma (TCGA, Firehose Legacy) | 0.0108 | | 13/478 |
| Head and Neck Squamous Cell Carcinoma | 0.633 | | 12/488 |
| Kidney Renal Clear Cell Carcinoma (TCGA, Pan-Cancer Atlas) | 0.307 | | 2/352 |
| Liver Hepatocellular Carcinoma | 0.698 | | 26/348 |
| Ovarian Serous Cystadenocarcinoma (mutation + high mRNA) | 0.0058 | | 16/201 |
| Ovarian Serous Cystadenocarcinoma (high mRNA only) | 0.0221 | | 10/201 |
| Pancreatic Adenocarcinoma | 0.634 | | 10/168 |
| Skin Cutaneous Melanoma | 0.494 | | 18/363 |
| Sarcoma | 0.380 | | 10/251 |
| Thyroid Carcinoma | 0.0260 | | 17/480 |
| Papillary Thyroid Carcinoma | 0.0438 | | 15/388 |
| Uterine Corpus Endometrial Carcinoma | 0.412 | | 25/507 |
| Uterine Carcinosarcoma (amplification + high mRNA) | 0.0071 | | 6/56 |

**Table S11. Microarray datasets from GEO database associated with *APOC2* expression analysis**

| **Cancer Type** | **GEO Accession** | **Tissue** | **Platform** |
| --- | --- | --- | --- |
| Glioblastoma | GSE4536 | Glioblastoma | Affymetrix Human Genome U133 Plus 2.0 Array |
| Glioblastoma | GSE2223 | Glioblastoma | SHFK |
| Glioblastoma | GSE2223 | Anaplastic Oligoastrocytoma | SHFK |
| Breast Cancer | GSE1477 | Invasive Lobular Breast Carcinoma | Protein Design Labs Hu03 Custom Affymetrix GeneChip Array |
| Breast Cancer | GSE1477 | Invasive Ductal Breast Carcinoma | Protein  Design Labs Hu03 Custom Affymetrix GeneChip Array |
| Breast Cancer | GSE3744 | Ductal Breast Carcinoma | Affymetrix Human Genome U133 Plus 2.0 Array |
| Lymphoma | GSE2350 | Lymphoma's Centroblastic Lymphoma | Affymetrix Human Genome U95A Array;  Affymetrix Human Genome U95 Version 2 Array |
| Lymphoma | GSE2350 | Diffuse Large B Cell Lymphoma | Affymetrix Human Genome U95A Array;  Affymetrix Human Genome U95 Version 2 Array |
| Lymphoma | GSE2350 | Burkitt's Lymphoma | Affymetrix Human Genome U95A Array;  Affymetrix Human Genome U95 Version 2 Array |
| Colorectal Cancer | GSE9348 | Colorectal Carcinoma | Affymetrix Human Genome U133 Plus 2.0 Array |
| Hypopharyngeal Cancer | GSE2379 | Head and Neck Squamous Cell Carcinoma | Affymetrix Human Genome U95A Array |
| Renal Tumor | GSE11151 | Papillary Renal Cell Carcinoma | Affymetrix Human Genome U133 Plus 2.0 Array |
| Renal Tumor | GSE11151 | Chromophobe Renal Cell Carcinoma | Affymetrix Human Genome U133 Plus 2.0 Array |
| Renal Tumor | GSE11151 | Clear Cell Renal Cell Carcinoma | Affymetrix Human Genome U133 Plus 2.0 Array |
| Melanoma | GSE7553 | Cutaneous Melanoma | Affymetrix Human Genome U133 Plus 2.0 Array |
| Melanoma | GSE7553 | Skin Squamous Cell Carcinoma | Affymetrix Human Genome U133 Plus 2.0 Array |
| Gastric Cancer | GSE19826 | Gastric Cancer | Affymetrix Human Genome U133 Plus 2.0 Array |

**Supplementary Figures:**

**Figure S1**

**
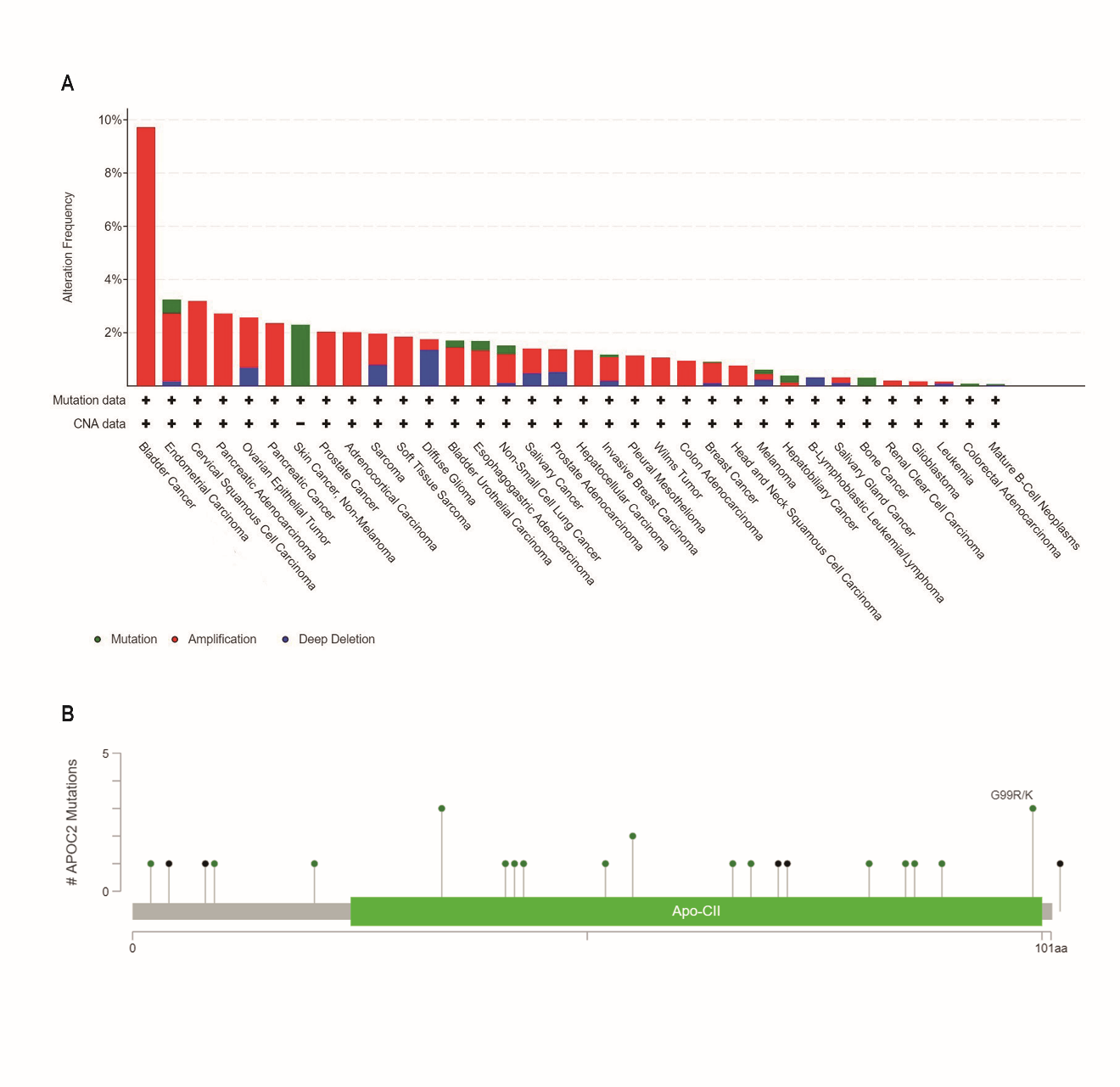
**

**Figure S1. Patterns of *APOC2* gene deregulation in cancer.** (A) Mutation and copy number alteration analysis of *APOC2* in different cancers determined by cBioPortal. The alterations include amplification (red), deep deletion (blue) and mutation (green). “+” indicates the existence of corresponding alteration type. “-” indicates lack of corresponding alteration type. (B) Mutation diagram of *APOC2* in cancer. The diagram presents the mutation sites and frequencies of *APOC2*. Green spots represent missense mutations. Black spots represent truncating mutations.

**Figure S2**


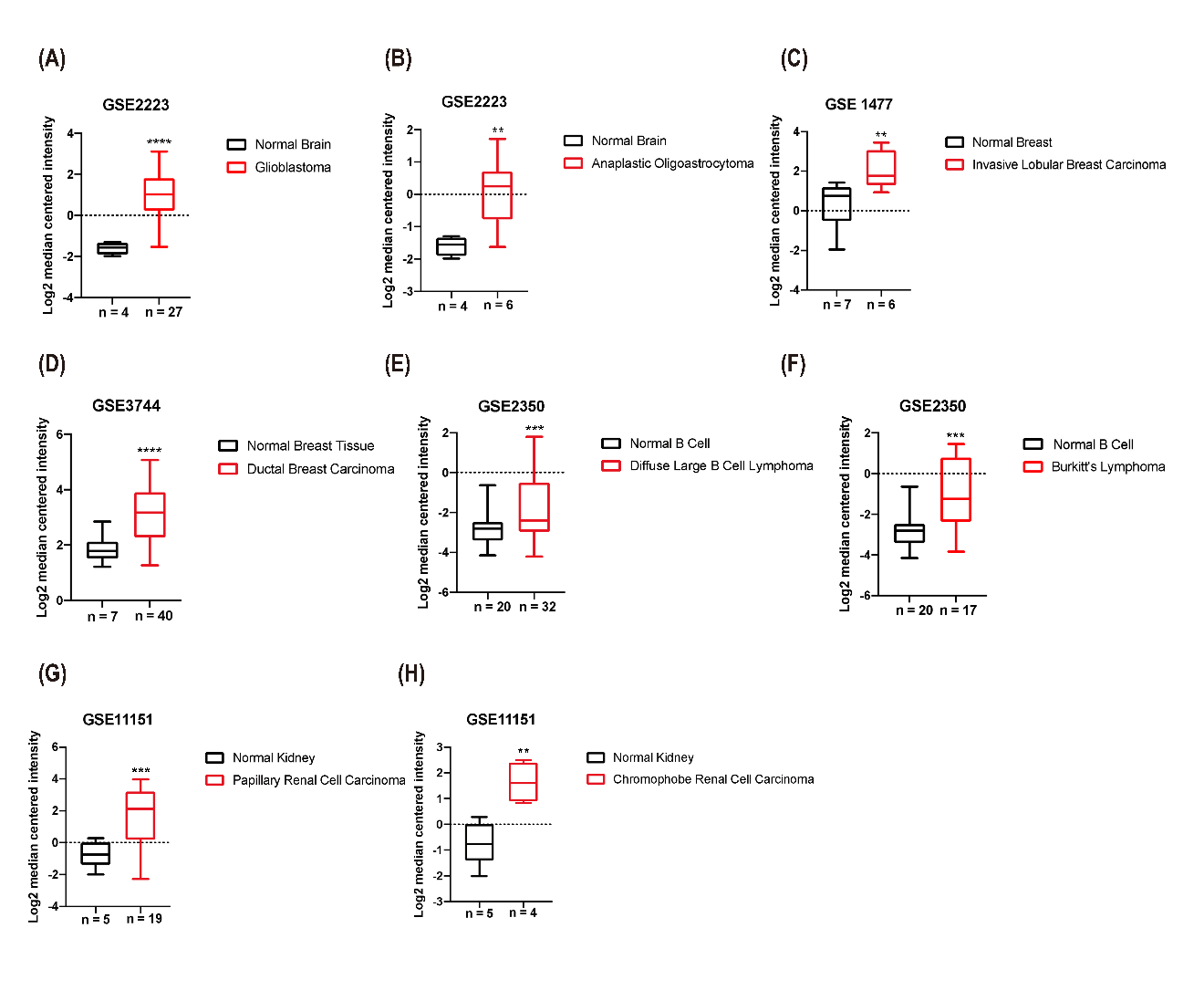


**Figure S2. Analysis of *APOC2* expression in different cancers**. *APOC2* expression raw

data was retrieved from the GEO database. The mean fold change was compared between

cancer group and healthy control in (A) glioblastoma, (B) anaplastic oligoastrocytoma,

(C) invasive lobular breast carcinoma, (D) ductal breast carcinoma, (E) diffuse large B

cell lymphoma, (F) Burkitt’s lymphoma, (G) papillary renal cell carcinoma, and (H)

chromophobe renal carcinoma. The differences between groups were analyzed by unpaired t-test. (****, P < 0.0001; ***, P < 0.001; **, P < 0.01)

**Figure S3**


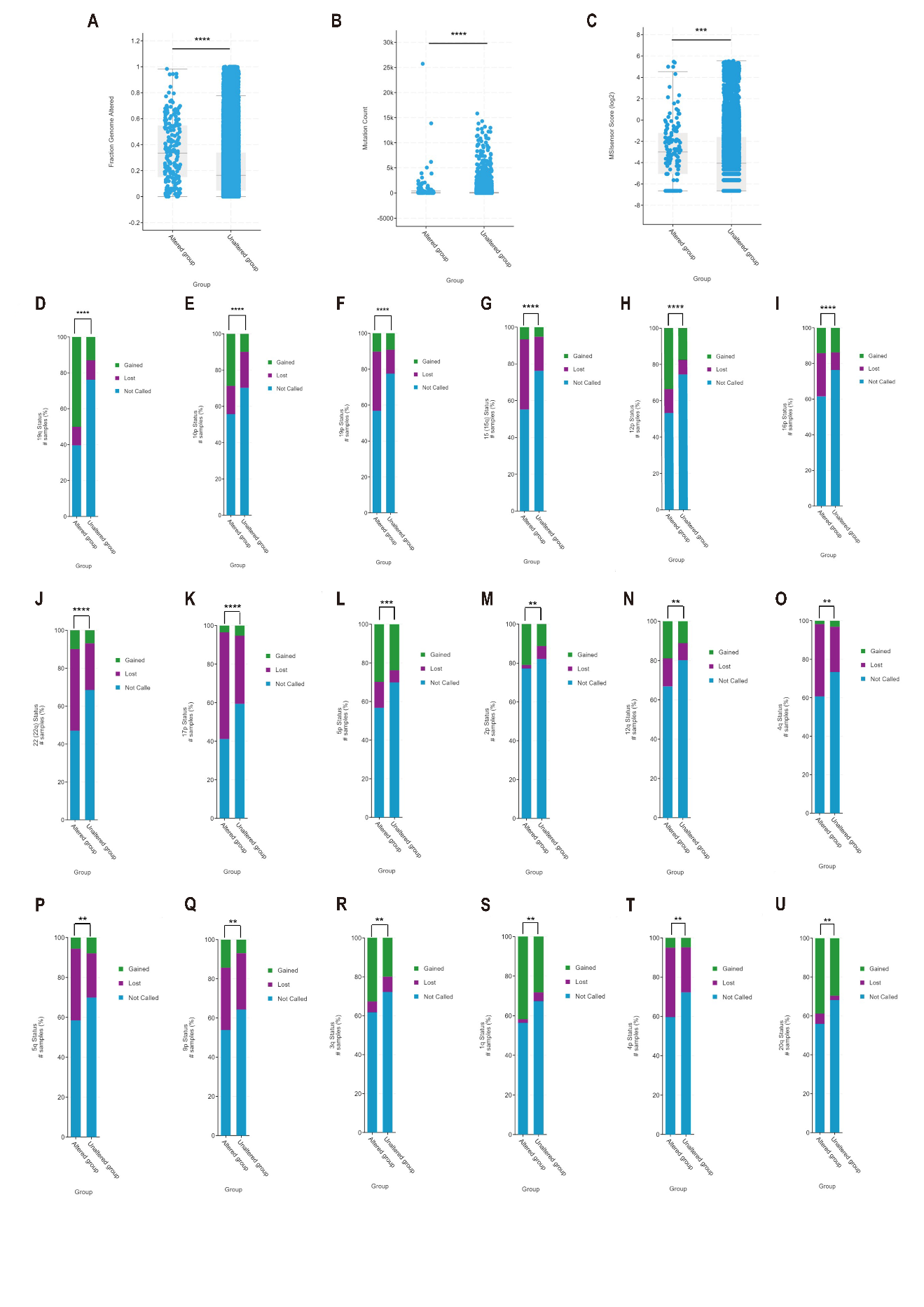


**Figure S3. Clinical attributes associated with alteration of *APOC2*.** (A) Fraction genome altered in *APOC2* altered and unaltered group. (B) Mutation count in *APOC2* altered and unaltered group. (C) Microsatellite instability (MSI) quantified by MSIsensor score in *APOC2* altered and unaltered group. The frequencies of chromosomal gain and lost in *APOC2* altered and unaltered groups were compared in chromosome (D) 19q, (E) 10p, (F) 19p, (G) 15q, (H) 12p, (I) 16p, (J) 22q, (K) 17p, (L) 5p, (M) 2p, (N) 12q, (O) 4q, (P) 5q, (Q) 9p, (R) 3q, (S) 1q, (T) 4p, and (U) 20q. *APOC2* altered groups included patient samples with *APOC2* mutation and copy number alterations. The differences between groups in panel (A), (B) and (C) were analyzed by Kruskal-Wallis test. The differences between groups associated with chromosomal gain and loss were calculated using Chi-squared test. (****, P < 0.0001; ***, P < 0.001; **, P < 0.01)

**Figure S4**


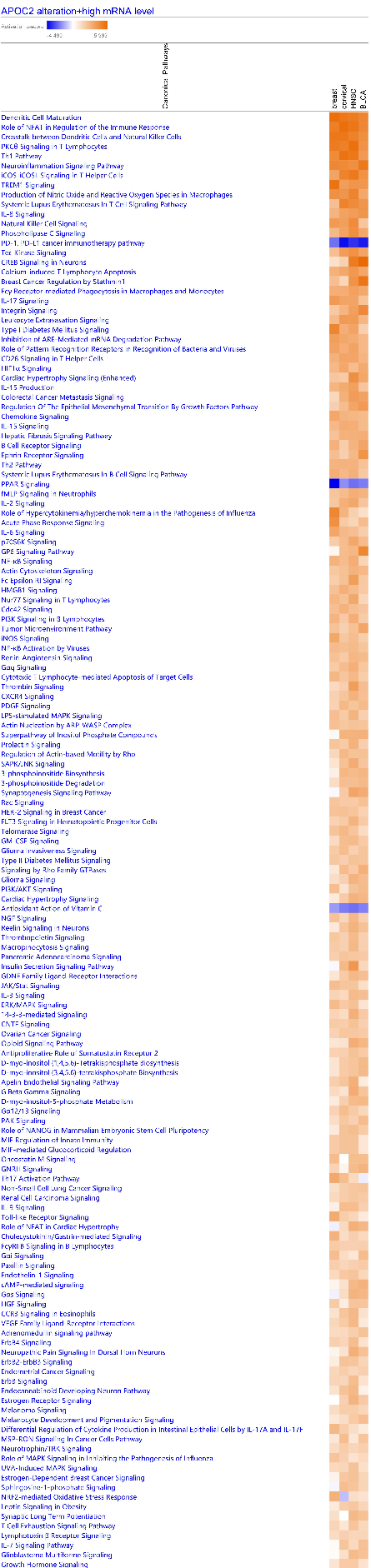


**Figure S4. Ingenuity pathway analysis associated with alteration of *APOC2*.** Four data sets were included in the ingenuity pathway analysis (IPA) in which genetic alterations including amplification, mutation and high mRNA level were found in *APOC2*. Gene expression log ratios of significantly altered genes in patients with *APOC2* alterations compared with patients without *APOC2* alterations were the input for the IPA. Pathways shown in orange are upregulated while pathways shown in blue are downregulated. Abbreviations: HNSC: head and neck squamous cell carcinoma, BLCA: bladder urothelial carcinoma.

**Figure S5**


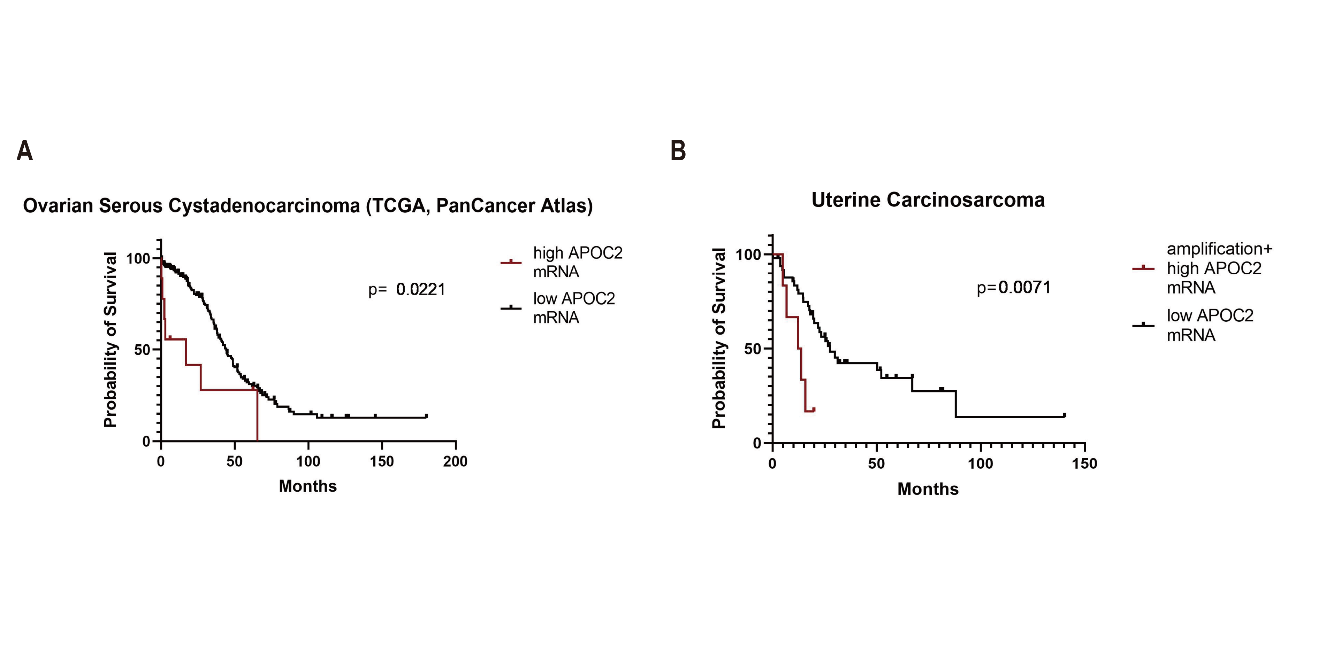


**Figure S5. Survival analysis for patients with different types of cancers associated with *APOC2* alteration and mRNA expression.** (A) Overall survival of ovarian serous cystadenocarcinoma patients with high (Z > 2) and low (Z < -2) *APOC2* mRNA level. (B) Overall survival of uterine carcinoma patients with *APOC2* amplification and high (Z > 2) mRNA level versus low (Z < -2) *APOC2* mRNA level. The differences between groups were analyzed by Mantel-Cox test.

**Supplementary references:**

1. Cerami E, Gao J, Dogrusoz U, Gross BE, Sumer SO, Aksoy BA, Jacobsen A, Byrne CJ, Heuer ML, Larsson E, Antipin Y, Reva B, Goldberg AP, Sander C, Schultz N. The cBio cancer genomics portal: an open platform for exploring multidimensional cancer genomics data. *Cancer Discov* 2012;2:401-4.
2. Gao J, Aksoy BA, Dogrusoz U, Dresdner G, Gross B, Sumer SO, Sun Y, Jacobsen A, Sinha R, Larsson E, Cerami E, Sander C, Schultz N. Integrative analysis of complex cancer genomics and clinical profiles using the cBioPortal. *Sci Signal* 2013;6:pl1.
3. Chandrashekar DS, Bashel B, Balasubramanya SAH, Creighton CJ, Ponce-Rodriguez I, Chakravarthi BVSK, Varambally S. UALCAN: A Portal for Facilitating Tumor Subgroup Gene Expression and Survival Analyses. *Neoplasia* 2017;19:649-658.
4. Davis S, Meltzer PS. GEOquery: a bridge between the Gene Expression Omnibus (GEO) and BioConductor. *Bioinformatics* 2007;23:1846-7.
5. Myklebost O, Williamson B, Markham AF, Myklebost SR, Rogers J, Woods DE, Humphries SE. The isolation and characterization of cDNA clones for human apolipoprotein CII. *J Biol Chem* 1984;259:4401-4.
6. Mak PA, Laffitte BA, Desrumaux C, Joseph SB, Curtiss LK, Mangelsdorf DJ, Tontonoz P, Edwards PA. Regulated expression of the apolipoprotein E/C-I/C-IV/C-II gene cluster in murine and human macrophages. A critical role for nuclear liver X receptors alpha and beta. *J Biol Chem* 2002;277:31900-8.
7. Shen Y, Lookene A, Nilsson S, Olivecrona G. Functional analyses of human apolipoprotein CII by site-directed mutagenesis: identification of residues important for activation of lipoprotein lipase. *J Biol Chem* 2002;277:4334-42.
8. Zdunek J, Martinez GV, Schleucher J, Lycksell PO, Yin Y, Nilsson S, Shen Y, Olivecrona G, Wijmenga S. Global structure and dynamics of human apolipoprotein CII in complex with micelles: evidence for increased mobility of the helix involved in the activation of lipoprotein lipase. *Biochemistry* 2003;42:1872-89.
9. Connelly PW, Maguire GF, Little JA. Apolipoprotein CIISt. Michael. Familial apolipoprotein CII deficiency associated with premature vascular disease. *J Clin Invest* 1987;80:1597-606.
10. Wilson CJ, Priore Oliva C, Maggi F, Catapano AL, Calandra S. Apolipoprotein C-II deficiency presenting as a lipid encephalopathy in infancy. *Ann Neurol* 2003;53:807-10.
11. Jiang J, Wang Y, Ling Y, Kayoumu A, Liu G, Gao X. A novel APOC2 gene mutation identified in a Chinese patient with severe hypertriglyceridemia and recurrent pancreatitis. *Lipids Health Dis* 2016;15:12.
12. Kuemmerle NB, Rysman E, Lombardo PS, Flanagan AJ, Lipe BC, Wells WA, Pettus JR, Froehlich HM, Memoli VA, Morganelli PM, Swinnen JV, Timmerman LA, Chaychi L, Fricano CJ, Eisenberg BL, Coleman WB, Kinlaw WB. Lipoprotein lipase links dietary fat to solid tumor cell proliferation. *Mol Cancer Ther* 2011;10:427-36.
13. Cao D, Song X, Che L, Li X, Pilo MG, Vidili G, Porcu A, Solinas A, Cigliano A, Pes GM, Ribback S, Dombrowski F, Chen X, Li L, Calvisi DF. Both de novo synthetized and exogenous fatty acids support the growth of hepatocellular carcinoma cells. *Liver Int* 2017;37:80-89.
14. Henderson F, Johnston HR, Badrock AP, Jones EA, Forster D, Nagaraju RT, Evangelou C, Kamarashev J, Green M, Fairclough M, Ramirez IB, He S, Snaar-Jagalska BE, Hollywood K, Dunn WB, Spaink HP, Smith MP, Lorigan P, Claude E, Williams KJ, McMahon AW, Hurlstone A. Enhanced Fatty Acid Scavenging and Glycerophospholipid Metabolism Accompany Melanocyte Neoplasia Progression in Zebrafish. *Cancer Res* 2019;79:2136-2151.
15. Breckenridge WC, Little JA, Steiner G, Chow A, Poapst M. Hypertriglyceridemia associated with deficiency of apolipoprotein C-II. *N Engl J Med* 1978;298:1265-73.
16. Wiebusch H, Nofer JR, von Eckardstein A, Funke H, Wahrburg U, Martin H, Köhler E, Assmann G. Electrophoretic screening for human apolipoprotein C-II variants: repeated identification of apolipoprotein C-II(K19T). *J Mol Med (Berl)* 1995;73:373-8.
17. Ueda M, Dunbar RL, Wolska A, Sikora TU, Escobar MDR, Seliktar N, deGoma E, DerOhannessian S, Morrell L, McIntyre AD, Burke F, Sviridov D, Amar M, Shamburek RD, Freeman L, Hegele RA, Remaley AT, Rader DJ. A Novel APOC2 Missense Mutation Causing Apolipoprotein C-II Deficiency With Severe Triglyceridemia and Pancreatitis. *J Clin Endocrinol Metab* 2017;102:1454-1457.
18. Johansen CT, Wang J, McIntyre AD, Martins RA, Ban MR, Lanktree MB, Huff MW, Péterfy M, Mehrabian M, Lusis AJ, Kathiresan S, Anand SS, Yusuf S, Lee AH, Glimcher LH, Cao H, Hegele RA. Excess of rare variants in non-genome-wide association study candidate genes in patients with hypertriglyceridemia. *Circ Cardiovasc Genet* 2012;5:66-72.
19. Pinilla-Monsalve GD, Lores J, Pachajoa H, López-Ponce de León JD, López A, Rodríguez-Rojas LX, Nastasi-Catanese JA. A Novel *APOC2* Mutation in a Colombian Patient with Recurrent Hypertriglyceridemic Pancreatitis. *Appl Clin Genet* 2020;13:63-69.
20. Röhrig F, Schulze A. The multifaceted roles of fatty acid synthesis in cancer. *Nat Rev Cancer* 2016;16:732-749.
21. Petan T, Jarc E, Jusović M. Lipid Droplets in Cancer: Guardians of Fat in a Stressful World. *Molecules* 2018;23:1941.
22. Sakurai T, Sakurai A, Vaisman BL, Amar MJ, Liu C, Gordon SM, Drake SK, Pryor M, Sampson ML, Yang L, Freeman LA, Remaley AT. Creation of Apolipoprotein C-II (ApoC-II) Mutant Mice and Correction of Their Hypertriglyceridemia with an ApoC-II Mimetic Peptide. *J Pharmacol Exp Ther* 2016;356:341-53.
23. Shachter NS, Hayek T, Leff T, Smith JD, Rosenberg DW, Walsh A, Ramakrishnan R, Goldberg IJ, Ginsberg HN, Breslow JL. Overexpression of apolipoprotein CII causes hypertriglyceridemia in transgenic mice. *J Clin Invest* 1994;93:1683-90.
